# Spicing Up Meat Preservation: *Cinnamomum zeylanicum* Essential Oil in Meat-Based Functional Foods—A Five-Year Review

**DOI:** 10.3390/foods13162479

**Published:** 2024-08-07

**Authors:** Raluca-Aniela Gheorghe-Irimia, Dana Tăpăloagă, Paul-Rodian Tăpăloagă, Oana-Mărgărita Ghimpețeanu, Laurențiu Tudor, Manuella Militaru

**Affiliations:** 1 Faculty of Veterinary Medicine, University of Agronomic Sciences and Veterinary Medicine Bucharest, 050097 Bucharest, Romania; raluca.irimia@fmv.usamv.ro (R.-A.G.-I.); ghimpe_marga@yahoo.com (O.-M.G.); donlorenzofmv@yahoo.com (L.T.); manuella.militaru@fmvb.usamv.ro (M.M.); 2 Faculty of Animal Productions Engineering and Management, University of Agronomic Sciences and Veterinary Medicine Bucharest, 011464 Bucharest, Romania; rodiantapaloaga@yahoo.com

**Keywords:** *Cinnamomum zeylanicum* essential oil, functional foods, meat preservation

## Abstract

Today, in the modern consumer era, we are facing a significant change in terms of preferences and behaviour. This tendency is not only a basic desire, but rather a significant social and cultural movement that exerts a tremendous influence on the food industry and correlated sectors. In this direction, food authorities and experts have thoroughly evaluated the practicality of employing natural preservation methods to enhance the quality and safety of foodstuffs, while preserving their nutritional and sensory attributes. Given this context, the development of meat products enhanced with *Cinnamomum zeylanicum* essential oil (CZEO) poses promising avenues, such as extended shelf-life due to its antimicrobial, antifungal, and antioxidant properties. CZEO also has many health benefits, rendering it as a promising ingredient in functional meat product formulations. Conversely, challenges such as higher associated costs, sensory interactions, and variability arise. Hence, the aim of this review is to offer a novel critical perspective on CZEO’s potential application as a functional ingredient in meat products formulations and to address the inherent associated challenges, based on the last five years of scholarly publications.

## 1. Introduction

From conventional methods to more contemporary techniques such as modified atmosphere packaging, irradiation, and the use of chemical preservatives, meat preservation techniques have historically undergone extensive changes. Despite notable progress, the meat industry is facing substantial hurdles, including but not limited to food safety and quality, shelf-life extension, and the sensory and nutritional value preservation. The challenges highlight the importance of using novel preservation techniques that not only extend the shelf life of products and adhere to regulations regarding food safety but can also aid in functional food products’ development [1,2,3,4,5,6,7,8].

In this direction, nowadays we are witnessing an evident change regarding consumers’ behaviour. They tend to be more interested in the possible health hazards associated to artificial additives, prompting an industrial transition towards natural alternatives. To endorse this phenomenon, numerous studies have investigated plant-derived phenolic molecules and other compounds that can influence food preservation and have important health benefits, thereby substantiating this transition to natural preservatives [1,2,3,4]. In this regard, plant-based essential oils (EOs) are being explored for their functional properties. They offer a potential alternative to synthetic preservatives, also being considered eco-friendly. EOs are classified as generally safe and non-bio-hazardous by the Food and Drug Administration [9,10].

Among the most often researched EOs in the realm of the food industry is *Cinnamomum zeylanicum* essential oil (CZEO). Its antioxidant, antimicrobial, and health associated properties render it as a potent natural preservative and health promoter [11,12]. The research shows that it may hinder the growth of microorganisms and prolong meat products’ shelf life, being a promising natural alternative to synthetic additives. These properties are attributed to its bioactive molecules such as cinnamaldehyde, eugenol, and linalool; these compounds are still being investigated for their other potential benefits [13,14]. Nevertheless, the application of EOs in the actual food system remains a significant challenge due to their primary hurdles, which include their volatility, inability to dissolve in water, and chemical instability when exposed to light, heat, moisture, and oxygen during the storage and processing of food [10,15].

Considering the aforementioned, the aim of this review is to thoroughly analyse and synthesise the research conducted in the last five years on the use of CZEO in meat industry applications. This review endeavours to offer a comprehensive analysis of the chemical composition, antimicrobial, antioxidant, and health-related properties, application methods, and effectiveness of CZEO in different meat products, providing a strong foundation for functional CZEO meat products’ development. In addition, the review will cover safety and regulatory factors, the economic and environmental effects, and future perspectives in the realm of meat preservation.

## 2. Chemical Composition of CZEO

**Figure 1:**
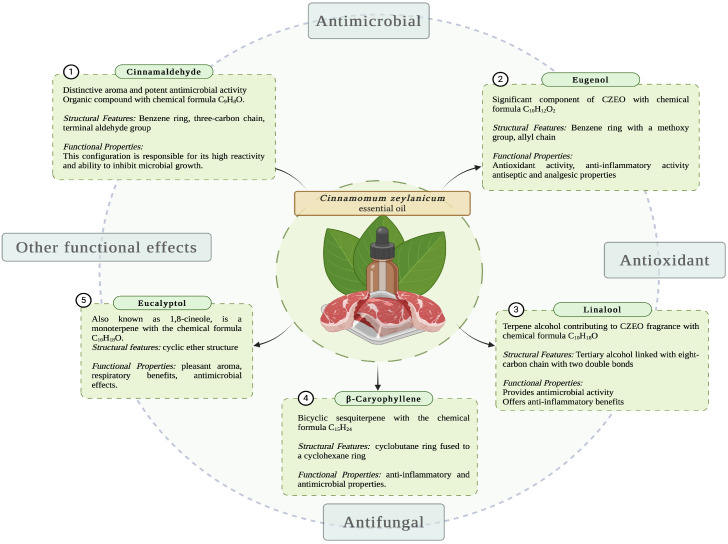
CZEO main chemical compounds (illustration made via BioRender.com).

*Cinnamomum zeylanicum* (synonymous with *C. verum*), a member of the *Lauraceae* family, is a plant that is renowned for its exceptional biological properties (Figure 1). It is abundant in India, Indochina, and Madagascar [16].

The primary constituent of CZEO is cinnamaldehyde (Table 1), which is well known for its crucial role in the distinct cinnamon aroma and also for its antibacterial, antioxidant, antifungal, and antiviral activities [16]. 

Cinnamaldehyde inhibits microorganisms’ proliferation by reacting with nitrogenous compounds, including nucleic acids and proteins. Therefore, the compound is essential for CZEO bioactivity [17].

Moreover, alongside cinnamaldehyde, CZEO has other essential compounds that enhance its biological efficiency (Figure 1). For example, eugenol which is a pale-yellow oil with a spicy scent (Table 1). Recent studies suggest that eugenol exhibits significant antioxidant properties by forming complexes with reduced metals. The compound effectively disrupts chain reactions by capturing active oxygen molecules, also being metabolised into a dimer that has the ability to reduce lipid peroxidation. Regarding its antibacterial effects, eugenol causes cell lysis by inducing the expulsion of protein and lipid from the cell membrane [18,19,20,21]. In addition, CZEO contains linalool, benzyl benzoate, and cinnamyl acetate, each serving a distinct function in terms of their biological properties. As an example, linalool is known for its ability to hinder microorganisms and alleviate inflammation and benzyl benzoate is a highly potent insecticide and acaricide.

Moreover, the efficacy of CZEO against microorganisms and its potential for medical applications are emphasised by the presence of carvacrol, thymol, and menthol (Table 1). A significant amount of research has been carried out on carvacrol and thymol, which are phenolic monoterpenes, in order to investigate their antibacterial properties. The addition of these compounds increases the versatility of CZEO as a natural product with a wide range of uses [22,23,24,25]. 

The synergistic interactions among these compounds have been examined, revealing that their combined action results in increased biological activity [26,27].

The CZEO chemical analysis revealed a rich variety of bioactive compounds that contribute to the EOs’ functional properties. Optimising the usage of CZEO in direct food matrix applications in the meat sector requires a thorough understanding of its chemical composition. Nevertheless, it is important for future research to focus on the standardisation and quality control of CZEOs to guarantee uniformity and safety across different applications.

**Table 1:** Chemical composition of CZEO.

EO	Extraction Method	Characterisation Method	Composition	Ref.
*Cinnamomum zeylanicum Nees*	The CZEO was extracted from leaves and stem. The extraction method was hydro distillation with water vapor for 4 h.	Gas chromatography (GC) with flame ionisation detector (FID) and gas chromatography–mass spectrometry (GC-MS)	Leaves: Benzyl benzoate (74.2%), α-phellandrene (6.9%), α-pinene (3.0%), linalool (2.7%), α-thujene (0.5%), camphene (1.0%), benzaldehyde (1.1%), myrcene (0.6%), *p*-cymene (1.6%), limonene (1.0%), 1,8-cineole (0.5%), terpinolene (0.6%), α-caryophyllene (0.7%), α-cadinene (1.1%), spathulenol (0.6%), others (4.0%) Stem: Cinnamaldehyde (31.0%), linalool (13.3%), benzyl benzoate (11.3%), cinnamaldehyde acetate (8.2%), α-thujene (0.4%), α-pinene (2.8%), camphene (1.0%), benzaldehyde (0.7%), myrcene (0.4%), α-phellandrene (2.3%), *p*-cymene (6.5%), α-terpinene (0.4%), limonene (1.6%), 1,8-cineole (1.2%), α-caryophyllene (3.7%), caryophyllene oxide (3.9%), others (10.4%)	[26]
*Cinnamomum zeylanicum*	The CZEO bark was extracted using the hydrodistillation method with a Clevenger type apparatus.	Gas chromatography–mass spectroscopy (GC-MS) method	Cinnamaldehyde, eugenol, α-pinene, eucalyptol, cinnamic acid, α-terpineole	[27]
*Cinnamomum zeylanicum*	The CZEO was extracted from its dried bark using a Clevenger-type apparatus for 3 h. The extracted oil was collected in clean glass vials, dried with anhydrous sodium sulfate, and stored at 4 °C until further analyses.	GC-MS (gas chromatography–mass spectrometry) and FTIR (Fourier transform infrared) spectroscopy	α-Pinene: 1.3% Benzaldehyde: 0.3% *p*-Cymene: 1.9% Limonene: 1.2% Eucalyptol: 5.4% γ-Terpinene: 0.4% Linalool: 7% Isoborneol: 0.8% (*E*)-cinnamaldehyde: 71.5% Eugenol: 4.6% β-Caryophyllene: 6.4% Acetic acid, cinnamyl ester: 0.5% α-Humulene: 1.7% δ-Cadinene: 1.4% trans-Calamenene: 0.7% Caryophyllene oxide: 0.5% - Benzyl benzoate: 0.5%	[11]
*Cinnamomum zeylanicum*	The CZEO was extracted from the stem bark of the plant using steam distillation. The plant originated from Sri Lanka.	Gas chromatography–mass spectrometry (GC-MS) and gas chromatography with flame ionisation detection (GC-FID)	(*E*)-Cinnamaldehyde: 77.42% Eugenol: 8.17% (*E*)-Cinnamyl acetate: 4.50% Benzaldehyde: 0.87% o-Cimene: 0.34% β-Phellandrene: 0.34% 1,8-Cineol: 1.42% Linalool: 3.5% α-Copaene: 1.91% (*E*)-Caryophylene: 1.1% - δ-Cadinene: 0.43%	[28]
*Cinnamomum zeylanicum* (cultivated cinnamon), *Cinnamomum capparu-coronde*, *Cinnamomum dubium*, and *Cinnamomum sinharajaense*	*Cinnamomum zeylanicum* leaf, stem-bark, and root bark from Sri Lanka.	Gas chromatography coupled to a flame ionisation detector and mass spectrometry detection	trans-Cinnamaldehyde, eugenol, camphor	[29]
*Cinnamomum zeylanicum*	The leaves of *Cinnamomum zeylanicum* were collected from three different altitudes in Uttarakhand. The method of extraction is not mentioned.	Gas chromatography/gas chromatography–mass spectrometry analysis	(*E*)-Cinnamaldehyde, (*E*)-cinnamyl acetate, linalool, phenyl propanoids, sesquiterpenes	[30]
*Cinnamomum zeylanicum (cinnamon)*	The essential oil was extracted from the bark of wild *Cinnamomum zeylanicum* grown in the green mountains of Oman.	Gas chromatography–mass spectrometry	Thirty compounds were identified, including the major constituents cinnamaldehyde (81.78%), bornyl acetate (5.33%), and cinnamyl acetate (2.82%)	[31]
*Cinnamomum zeylanicum (Blume)*	The barks of *Cinnamomum zeylanicum* were purchased from a local market in Turkey. The CZEO was extracted via hydrodistillation for 3 h using a Clevenger apparatus.	GC-MS analysis	(*E*)-Cinnamaldehyde (CAL), (*E*)-cinnamyl acetate (CAS), and 20 other minor components	[32]
*Cinnamomum zeylanicum*	The CZEO was extracted from bark samples collected from the market of Basrah governorate. Essential oils were then extracted using a Clevenger apparatus through water distillation at 100 °C for four hours. The recovered oil was dried with anhydrous sodium sulfate and stored in a dark glass container.	Gas chromatography–mass spectrometry (GC-MS)	Cinnamaldehyde (2-Propenal, 3-phenyl-) (46.46%) 9-Methoxybicyclo [6.1.0]nona-2,4,6-triene (31.31%) alpha-Muurolene (7.14%) tau-Muurolol (1.34%) Copaene (1.63%) Benzaldehyde (0.10%) Benzaldehyde dimethyl acetal (0.11%) Benzenepropanal (0.04%) Borneol (0.02%) Cinnamaldehyde, (E)- (0.23%) 1,4-Methano-1H-indene, octahydro-4-methyl-8-methylene-7-(1-methylethyl)- (0.57%) 2-Propenoic acid, 3-(2-hydroxyphenyl)- (0.29%) 2-Propen-1-ol, 3-phenyl-, acetate, (*E*)- (0.21%) 1-Naphthalenol,1,2,3,4,4a,7,8,8a-octahydro-1,6-dimethyl-4-(1-methylethyl)- (1.18%) Naphthalene, 1,2,3,5,6,8a-hexahydro-4,7-dimethyl-1-(1-methylethyl)- (5.95%) Naphthalene, 1,2,3,4,4a,7-hexahydro-1,6-dimethyl-4-(1-methylethyl)- (0.69%) Benzene, 1-methyl-4-[(1-methylethylidene)cyclopropyl]- (1.04%) Caryophyllenyl alcohol (0.15%) 1-Hydroxy-1,7-dimethyl-4-isopropyl-2,7-cyclodecadiene (0.09%) Illudol (0.09%) Epiglobulol (0.32%) Cubenol (0.38%) 1H-Cycloprop[e]azulene, decahydro-1,1,7-trimethyl-4-methylene- (0.06%) Cycloheptane, 4-methylene-1-methyl-2-(2-methyl-1-propen-1-yl)-1-vinyl- (0.22%) 2-Butanone, 4-(2,6,6-trimethyl-2-cyclohexen-1-ylidene)- (0.07%) 2,5,5,8a-Tetramethyl-4-methylene-6,7,8,8a-tetrahydro-4H,5H-chromen-4a-yl hydropero (0.07%)	[33]
*Cinnamomum zeylanicum (cinnamon)*	The CZEO was extracted from leaves obtained from a local market using the steam distillation method.	LC-HRMS, GC-MS, and GC-FID	(*E*)-Cinnamaldehyde: 72.98% Benzyl benzoate: 4.01% trans-Cinnamyl acetate: 3.36% α-Pinene: 1.00% Camphene: 0.34% β-Pinene: 0.38% Phellandrene: 0.70% *p*-Cymene: 1.48% Linalool: 1.80% α-Terpineol: 0.48% *Z*-Cinnamaldehyde: 1.10% Safrole: 1.18% Eugenol: 1.48% α-Copaene: 0.77% β-Caryophyllene: 3.45% α-Humulene: 0.63% Acetyleugenol: 1.58% (−)-Caryophyllene oxide: 0.98% Hispidulin: 9.98 mg/L oil Herniarin: 7.82 mg/L oil - Apigenin: 6.61 mg/L oil	[34]
*Cinnamomum zeylanicum Blume*	The CZEO extraction method used was steam distillation, optimised using response-surface methodology. The CZEO originated from a local market in Sfax, Tunisia.	Gas chromatography–mass spectrometry (GC-MS) using an Agilent-Technologies Model 6890N network gas chromatograph system with a flame ionisation detector and HP-5MS capillary column	Benzaldehyde: 0.23% 1,8-Cineole: 3.19% γ-Terpinene: 0.16% Linalool: 0.30% Camphenilol: 0.02% Borneol: 0.31% Cyclohexene: 0.74% α-Pinene: 2.60% α-Terpinene: 0.38% Cinnamaldehyde: 77.34% trans-Caryophyllene: 0.13% Eugenol: 0.02% Hydrocinnamic acid-2,3-13C2: 0.13% trans-Cinnamyl acetate: 4.98% Coumaric acid: 1.79% Propenoic acid: 0.75% δ-Cadinene: 0.14% Caryophyllene oxide: 0.17% Naphthalenol: 0.05% Hexadecanoic-d31: 0.37% 9-Octadecenoic acid: 1.32% Phthalic acid: 0.72% - 1,4-Benzenedicarboxylic acid: 3.55%	[12]

## 3. Bioactive Properties of CZEO in Meat Product Innovation

**Figure 2:**
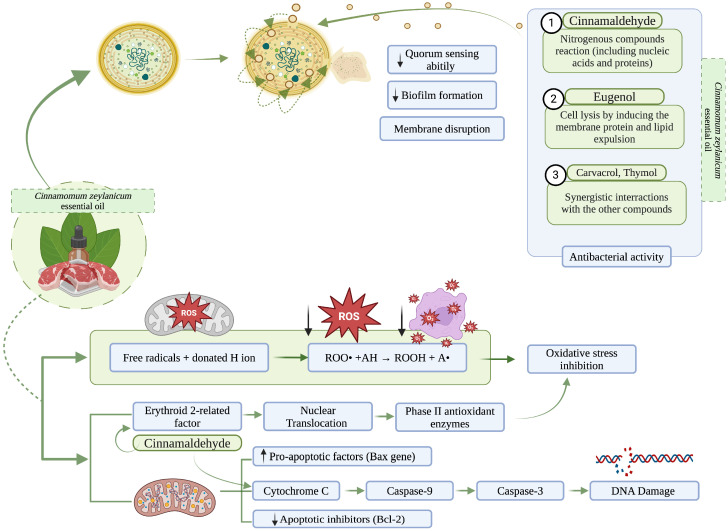
CZEO antimicrobial and antioxidant mechanism of action (illustration made via BioRender.com) [16,38].

Currently, multiple studies have demonstrated CZEO’s efficacy against a wide range of bacterial species and meat spoilage microorganisms (Table 2) [34,35,36]. For instance, El-Hack et al. (2020) underscored CZEO’s potent antibacterial properties, demonstrating its efficacy against bacteria such as *Parahemolyticus*, *Staphylococcus epidermis*, *Enterococus faecalis*, *Pseudomonas aeruginosa*, *Salmonella* sp., *Staphylococcus aureus*, and *Escherichia coli* [37]. Similar findings were observed by Saad et al. (2019) that determined the EOs’ efficacy against bacterial agents such as *Staphylococcus aureus* and *E. coli* and sensory properties of minced meat enriched with CZEO during cold storage at 4 °C, for five consecutive days. Their results showed a high antibacterial efficiency in CZEO-treated samples and improved sensory properties [14]. Behbahani et al. (2020) also observed CZEO’s efficiency against *Listeria innocua*, *Staphylococcus aureus*, *Bacillus cereus*, *Escherichia coli*, *Pseudomonas aeruginosa*, and *Salmonella typhi* and its mechanism of action by scanning electron microscopy [11]. The study concluded that the EO exhibited its antibacterial effect by disrupting the cell membrane and promoting the release of intracellular compounds. Other investigations have demonstrated that the EO can effectively impede microbial respiration in addition to increasing the permeability of the plasma membrane. Its properties may also be the consequence of the hydrophilic properties of the bacterial cell wall (Figure 2). Conversely, it was observed that its antimicrobial mechanism of action is pathogen-dependent [14,34,36,38].

The enhanced antimicrobial properties should also be an important target for functional food products’ development. One of the most important aspects is its efficiency against many pathogens that can impair human health. For example, Mutlu et al. (2023), observed that CZEO disrupted bacterial cell integrity and inhibited essential metabolic pathways in drug-resistant *Helicobacter pylori* strains [34]. Askari et al. (2023) studied the effect of CZEO on oral pathogens in humans. The review concluded that the antibacterial properties of the EO are comparable with those of chlorhexidine gluconate, ciprofloxacin HCl, or metronidazole. Remarkably, it was observed that CZEO inhibited cell division, adenosine triphosphate (ATP)-ase activity, biofilm formation, membrane porin, and lipid profile change through anti-quorum sensing effects [17]. In a similar vein, a topical problem for global public health is antimicrobial resistance. In this regard, CZEO’s mechanism of action against pathogens and its minimal risk of resistance in relation to its chemical composition renders it as a possible natural antimicrobial agent [38].

CZEO is also well known for its potent antioxidant properties, which have been extensively researched over the years (Table 3) [35]. In this regard, Behbahani et al. (2020) found the DPPH-RS (2,2-Diphenyl-1-picrylhydrazyl—radical scavenging) activity was 71.12 ± 0.77%, indicating its capacity to neutralise DPPH free radicals through either hydrogen atom or electron donation mechanisms. Similarly, the β-carotene bleaching assay determined that CZEO had a high inhibitory effect (63.08 ± 0.81% activity against β-carotene). The study concluded that CZEO’s antioxidant activity is attributed to its high phenolic and bioactive compounds [11]. The results are in line with the findings observed in a study conducted by Kallel et al. (2019). The experimental design used in vitro models in comparison with BHT (butylated hydroxytoluene) and vitamin C. In this direction, the observations included a phosphomolybdenum potency of 108.75  ±  32.63 mg of essential oil/equivalent to 1 mg of vitamin C antioxidant power, 21.3% DPPH activity, and 55.2% H_2_O_2_ activity, indicating an efficiency that is comparable with the positive controls. Using a different experimental approach, Teles et al. (2020) conducted a comparative analysis of the antioxidant activity of EOs derived from commonly used spices in the Brazilian food industry. The antioxidant activity of CZEO was determined by the adapted ABTS method [2,2-azinobis-(3-ethylbenzothiazoline-6-sulfonic acid)]. The effective concentration 50% (EC50) (μg mL^−1^) in CZEO was 215.93 and the % ABTS inhibition (50 μg mL^−1^) was 11.11, suggesting lower antioxidant properties compared with other EOs. On the other hand, the discrepancies can be due to EOs’ variability depending on factors such as the geographical origin, botanical source, harvesting time, or seasonal variations [46].

In terms of the mechanism of action, studies suggested that the active compounds of cinnamon possess the ability to donate a hydrogen atom to free radicals. Once the hydrogen atom is acquired, the free radicals become stable and alleviate any more oxidative stress on the cell (Figure 2) [38,47].

A noteworthy aspect is also the fact that CZEO is shown to have antifungal and antimycotoxigenic properties [48]. In this regard, research conducted by Perczak et al. (2019) found that eight essential oils, including CZEO, effectively reduced group B trichothecenes concentration levels ranging from 95.51 to 100% [49].

In addition to its antimicrobial and antioxidant properties, numerous studies also emphasised CZEO’s wide range of therapeutic properties (Table 4). Notably, in a study conducted by Thakur et al. (2021) the anti-inflammatory, antiproliferative, antidiabetic, wound healing, HIV/AIDS aid, antianxiety and antidepressant, and anti-Parkinson activities were underlined [50]. Similarly, CZEO’s antiproliferative properties were observed by Behbahani et al. (2020). The essential oil (EO) had a dose-dependent antiproliferative impact on adipose-derived mesenchymal stem cells (AT-MSCs), which was shown to be enhanced as its concentration increased up to 200 mg·mL^−1^. The IC_50_ for CZEO’s antiproliferative effect on AT-MSCs was found to be 3.51 mg·mL^−1^. The same effect was also observed in different cell lines, such as F2408 (normal rat fibroblasts) and 5RP7 (H-ras active-rat fibroblasts) [11]. Cappeli et al. (2023) also observed that CZEO determined metastatic melanoma cell (M14) inhibition, cycle disruptions, reactive oxygen species, and Fe(II) elevation and the depolarisation of the mitochondrial membrane [51]. Correspondingly, it was found that CZEO can affect the mevalonate metabolism pathway or induce cellular apoptosis [11].

In a similar context, an extensive body of research underlines the CZEO’s antidiabetic effect. In this regard, a study performed by Mohammed et al. (2020) on rats observed that 200 and 400 mg/kg b.w. of CZEO alleviated the glucose, insulin, amylase, superoxide dismutase, glutathione, and hepatic plasma malondialdehyde levels [52]. In the same species, a dose of 20 mg/kg b.w. determined insulinotropic effects characterised by enhanced glucose absorption via GLUT4 receptors and improved activity of pyruvate kinase and phosphoenolpyruvate carboxykinase [53]. In humans, a 400 mg/day CZEO dose determined an improvement in glucose and insulin levels, along with quality-of-life measurements [54]. In mice, a 0.02% cinnamaldehyde-supplemented diet enhanced the tonus of the aorta and restored the increased levels of renal markers to their normal state. The treatment also improved glomerular fibrosis, the findings suggesting a protective effect against vascular dysfunction through the inhibition of oxidative stress by activating the Nrf2 signalling pathway [55].

According to the presented research, CZEO has the potential to improve food safety, increase its shelf life, and potentially provide health benefits. Further study is required to investigate the mechanisms and applications of these bioactive properties in order to strengthen CZEO’s position in new food preservation technologies and health-promoting formulations.

**Table 4:** Summary of health-related functional properties of CZEO.

EO	Therapeutic Properties	Future Research	Ref.
*Cinnamomum zeylanicum*, Sri Lanka	Antioxidant and protective efficacy, free radical scavenging activity, reducing oxidative stress-induced complications	More studies are needed at the molecular level to understand the pathophysiology of clinical conditions related to oxidative stress.	[56]
*Cinnamomum zeylanicum*, Sfax, Tunisia	Antiproliferative effects, antioxidant properties	Composition–effect–mechanism–dose relationship investigation of CZEO using more in vitro and in vivo bioassay tests.	[12]
*Cinnamomum zeylanicum L*.	Tumour volume and incidence reduction, apoptosis promotion, antiproliferative effects, antiangiogenic effects, antioxidant effects, and epigenetic regulation	Efficacy, dosage, and potential side effects of using plant foods for breast cancer chemoprevention in humans, through well-designed clinical trials. Therapeutic potential of cinnamon and other natural plant-derived compounds in more heterogeneous human breast cancer models that can reflect the diversity of genotypes and phenotypes seen in clinical settings.	[57]
*Cinnamomum zeylanicum*	Antibacterial properties against extensively drug-resistant bacteria	In vivo investigations of CZEO to determine its effective ingredients for the synthesis of a new drug-resistant human pathogens antimicrobial agent.	[40]

## 4. Applications of CZEO in Meat Processing

For the meat industry, the concept of using meat products for health improvement instead of merely for consumption offers a fresh prospect. The increasing and major impact of scientific understanding of the link between nutrition and health and the consumer approach towards their food decisions is evident. This approach seeks to promote optimal health by enhancing wellbeing and reducing the risk of disease through the production of a superior food supply. This strategy is a pragmatic and ground-breaking approach to preventative healthcare [58,59].

It is widely recognised that consuming a large amount of meat is associated with increased oxidative stress, mainly due to the creation of oxidised substances such as 4-hydroxy-nonenal, oxysterols, malondialdehyde, and protein carbonyls. These molecules have the potential to cause oxidative damage. Aside from conventional presentations, the meat industry has the opportunity to explore different avenues. This includes regulating the composition of raw meat through dietary adjustments and manipulating processed meat by using a range of functional ingredients [58,59,60].

In relation to the dietary strategy, Abd-El-Hack et al. (2020) found that using CZEO as a supplement in poultry feed might yield advantageous outcomes in terms of performance (improved body weight and weight gain), hypocholesterolaemia effects, antioxidant activity, immunity, and microbiological aspects. The study highlighted the potential use of this approach as a substitute for antibiotics in the poultry industry, offering increased safety in terms of health, environment, and economics [37]. Correspondingly, a study performed by Yang et al. (2019) found that administering a dosage of 400 mg/kg body weight over in 42-day-old broiler chicks resulted in an enhanced immune response, reduced levels of *E. coli*, and increased *Lactobacillus* and *Bifidobacterium* in the cecum [61]. In a similar direction, Torrechilas et al. (2021), tested the effect of different clove and CZEO concentrations (450 mg/kg and 880 mg/kg) on meat quality (pH, lipid oxidation, shear force, colour) and consumers’ acceptability in young, crossbred bulls. The CZEO treatment influenced (*p* < 0.05) the instrumental meat quality attributes and had no effects on the sensory or visual acceptability (*p* > 0.05). The study concluded that the use of EOs can be a natural alternative for reducing lipid oxidation [62]. The presented studies are of crucial importance regarding future advancements in other animal diet formulations.

**Figure 3:**
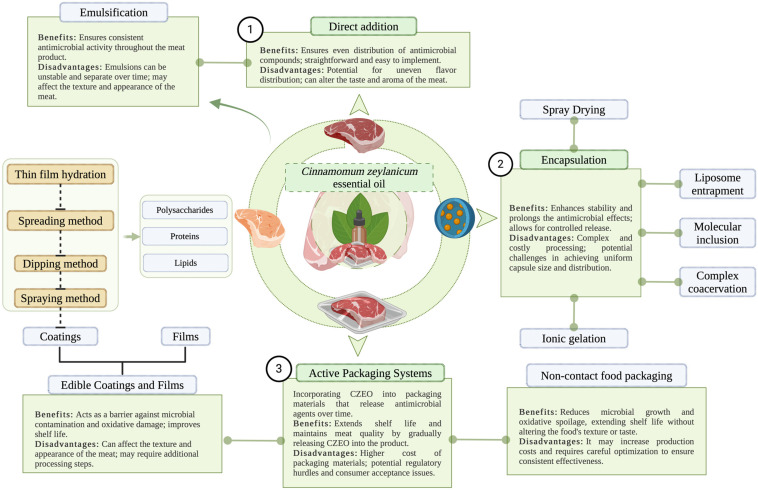
Different application methods of CZEO in meat products (illustration made via BioRender.com).

Regarding CZEO’s application in different meat product formulations, there are multiple documented techniques that can be employed (Figure 3, Table 5). The most popular approach in experimental designs is direct application. Along these lines, Mounika et al. (2023) tested three concentrations of CZEO (control, 0.2 µl/g, 0.4 µl/g, 0.6 µl/g) in refrigerated pork sausages stored 4 °C for 15 days. The study concluded that the 0.4 µl/g CZE supplementation had the most potent preservative effect [63]. Hussain et al. (2021) investigated the preservation properties of four different concentrations of CZEO (control, 0.01%, 0.025%, 0.05%, and 0.5%) in ground lamb meat. The samples were kept at a temperature of 4 °C for 16 days. Upon assessing the antibacterial impact, thiobarbituric acid reactive components, pH values, Chroma values, and relative concentration of oxymyoglobin, it was determined that CZEO 0.025% and 0.05% had a superior preservation effect on the quality of lamb meat during storage [64]. A similar experimental approach was also used by Zhang et al. (2019). Their study investigated the effect of two CZEO concentrations (control, 0.1%, and 0.5%, *v*/*w*) in fresh Italian-style sausages on different safety and quality traits. It was concluded that the treatments lowered the TBARS values (thiobarbituric acid reactive substances), b*value, biogenic amine contents, aerobic, and *Enterobacteriaceae* counts. Additionally, a dose-dependent effect of CZEO was observed, with the 0.5% treatment having superior results [65].

Another emerging CZEO application approach is the use of edible packaging, such as coatings and films [66,67]. Edible coatings are gaining popularity due to their ecologically advantageous properties and their capacity to carry active ingredients, distinguishing them from other forms of packaging. The use of an edible coating can mitigate the impact of EOs on the flavour of the product and extend the duration of their effects through a slow-release mechanism. This successfully enhances the utilisation of EOs in food [68]. EO edible coatings are mostly produced using three key groups of basic materials: polysaccharides, proteins, and lipids. Pertaining to this, Raeisi et al. (2019) incorporated CZEO and *Rosmarinus officinalis* EO (REO) into an alginate coating to enhance the chemical and sensory characteristics of chicken meat. Apart from the undeniable efficiency of CZEO, the study determined that REO was more potent as a preservative. On the other hand, a synergistic interaction of CZEO with REO was observed. The EOs’ blend exhibited higher performances in comparison with separate use. In this direction, the total volatile basic nitrogen (TVB-N) values (32.00 ± 1.48 mg/100 g), trimethylamine nitrogen (5.03 ± 0.5 mg/100 g), and total carbonyl contents (1.27 ± 0.13 nmol/mg) were lower compared to the other investigated treatments. Another critical detail is that both EOs were more efficient compared with the synthetic preservative used nowadays in the food industry—butylated hydroxyanisole (BHA). The study concluded the functional alginate-sodium coating increased the chicken meat shelf-life, hence demonstrating its potential as an effective food preservation method [69]. In the same direction, Mouhoub et al. (2022) examined the feasibility of producing chitosan-based biopolymer films with the inclusion of EOs. The study determined that the tested EOs, including CZEO, showed values of antibiofilm-forming activity ranging from 79.43% to 99.33% at 1 µL/mL concentrations, when adsorbed onto chitosan film. The results suggest that CZEO exhibits promising avenues as a component in biodegradable food packaging when mixed with chitosan [70].

A different approach is the use of active packaging materials that are not in contact with foods [71]. Prospective findings are being shown in this direction, such as the ones indicated by Ali et al. (2021). Researchers created a self-adhesive membrane using gum arabic that was modified with CZEO. This membrane was found to extend the shelf-life of food and also had antibacterial properties when used in a different food matrix [72]. Similarly, Songtipya et al. (2021) developed a novel natural rubber pressure sensitive adhesive patch supplemented with CZEO and xyloglucan for preserving a bakery product. The results indicated promising prospects for future research in meat industry applications [73].

A novel technique researched in the meat industry is CZEO encapsulation. Encapsulation entails encasing bioactive compounds, such as antioxidants, enzymes, polyphenols, and micronutrients, within protective wall materials prior to introducing them into a system. This method offers safeguarding and regulated dispensation for sensitive compounds [74]. Encapsulation techniques effectively overcome the limitations in the functionality of CZEO by protecting its bioactive compounds from degradation reactions, enhancing their solubility and stability even in unfavourable environmental conditions, increasing their bioavailability, hiding undesirable characteristics, enabling controlled release, and ultimately enhancing their biological effects. Several methods have been used to encapsulate CZEO, including spray drying, coacervation, precipitation, freeze-drying, ionic gelation, ultrasonication, and molecular inclusion. Every methodology possesses its fundamental principles, efficient methodological parameters, benefits, drawbacks, restrictions, and prospective applications (Table 5, Figure 3) [75,76,77,78,79,80]. Concerning this matter, Kean et al. (2022) studied the effect of different CZEO microcapsules on the physical and antimicrobial traits of minced chicken samples during refrigerated storage. The results indicated that the freeze-drying method with a 27% CZEO concentration had the highest solubility and encapsulation efficiency along with the lowest surface oil content. Additionally, the study concluded that the CZEO microcapsules had a significant effect on impeding bacterial growth [81]. Similarly, Dghais et al. (2022) conducted a study regarding the efficiency of CZEO and curcuma essential oil nanoemulsions as natural preservatives in beef meat. The antimicrobial and antioxidant test results indicated enhanced effects in nanoemulsion CZEO formulations (d3,2 = 89 nm and PDI = 0.32) compared to direct EO application. On the other hand, when applied onto the meat matrix, both treatments inhibited microbial growth, methaemoglobin, and lipid oxidation. Despite the similar results, it is worth noting that the nano-encapsulation method with Tween 80 possessed improved in vitro preservative properties [82].

The presented findings underscore the positive impact of CZEO in meat preservation as a promising alternative to synthetic preservatives. These results not only validate its functional properties but also highlight its potential to be applied using various methodologies with similar positive outcomes. On the other hand, future research is needed on optimising the formulation and dosage while ensuring the consumers’ acceptance and safety. Additionally, long-term shelf-life studies under various storage conditions are imperative to determine the stability and effectiveness of CZEO.

**Table 5:** Summary of CZEO application in meat products.

Species	Product Type	EO	Application Method	Outcome Measured	Main Findings	Advantages and Disadvantages	Ref.
POULTRY	Meat (broiler chicks and Japanese quail)	*Cinnamomum zeylanicum*	Dietary	Poultry performance, carcass traits, meat quality, hypocholesterolaemic effect, antioxidant activity, immunity, microbiological effect	CZEO decreased abdominal fat and cholesterol, increased the meat water holding capacity, and decreased the meat cholesterol. CZEO can be used as an alternative to antibiotics in poultry.	• Advantages Beneficial effects on cholesterol levels, antioxidant activity, antimicrobial properties, and digestive function. Antimicrobial and insecticidal properties. Ability to improve feed efficiency and growth performance by enhancing the immune system, gut microbiome, and digestive enzymes, as well as having antioxidant, antibacterial, and antiviral properties. • Disadvantages Inconsistent effects on feed intake and feed conversion ratio, with some studies finding no significant impact. Potential to decrease the water intake.	[37]
	Chicken meat	*Cinnamomum zeylanicum* and *Rosmarinus officinalis*	Coating (alginate)	Peroxide value, TBARS, trimethylamine nitrogen, total volatile basic nitrogen, sensory quality	CZEO incorporated into alginate coating significantly improved the chicken meat chemical parameters and sensory parameters	• Advantages Significant improvement in preserving the chemical and sensorial quality of chicken meat compared to the control during refrigerated storage, shelf-life extension. • Disadvantages The paper does not mention any clear disadvantages but recommends further research to scale up and commercialise the coating technology for industrial applications.	[69]
	Minced chicken meat	*Cinnamomum zeylanicum*	Encapsulation	Powder recovery, product quality, encapsulation efficiency, solubility, surface oil content, antimicrobial effect	The CZEO microcapsules significantly decreased the bacterial growth in minced chicken meat samples during chilled storage for 12 days.	• Advantages Freeze-drying has the highest encapsulation efficiency (92.3% to 95.2%) and the lowest surface oil content, being the most suitable encapsulation method. Freeze-drying with a 27% oil concentration has a higher solubility and encapsulation efficiency compared to the other methods. • Disadvantages Spray-drying has a much lower powder recovery compared to the other methods. Higher oil concentrations (for any encapsulation method) result in a lower powder recovery and reduced solubility.	[81]
LAMB	Meat	*Cinnamomum zeylanicum*	Coating (*Malva sylvestris* seed mucilage)	Antioxidant effects, antimicrobial effects	CZEO had antioxidant and antimicrobial effects on lamb meat slices during the experimental period (10 days, 4 °C).	• Advantages: Antioxidant and antimicrobial effects on lamb meat slices during refrigerated storage. • Disadvantages: Not mentioned	[83]
	Ground meat	*Cinnamomum zeylanicum*	Direct application	Microbial populations (log CFU/g), TBARS values, pH values, colour metrics (L^⁎^, a^⁎^, R630/580, Chroma), oxymyoglobin content	CZEO at 0.025% and 0.05% concentrations reduced microbial populations, lowered TBARS and pH values, and enhanced colour stability and oxymyoglobin content during storage at 4 °C.	• Advantages: Improved microbial safety and extended shelf-life due to the antimicrobial properties. Reduced lipid oxidation. Enhanced colour stability and retention, making the product more visually appealing. • Disadvantages: Higher costs of using cinnamon bark oil. Possible impact on the product flavour that may not be universally accepted by consumers.	[64]
BEEF	Minced beef	*Cinnamomum verum*	Direct application	Cinnamon leaf essential oil yield (%), cinnamaldehyde concentration (% area), antibacterial activity, microbiological efficiency (total viable count in CFU/g)	CZEO inhibited bacterial growth, particularly Gram-positive bacteria; and after 21 days of storage at 4 °C, the total viable count of minced beef with essential oil at 1.2% (*v*/*v*) was lower than 10^6^ CFU/g.	• Advantages Bacterial growth inhibition, particularly Gram-positive bacteria. Effective minced beef preservation (lower total viable count after refrigerated storage). Better yield and retention of functional properties with optimised extraction. • Disadvantages: Time-consuming and energy-intensive extraction process. Lower yield and higher rate of product degradation with traditional steam distillation.	[84]
	Minced beef	*Cinnamomum zeylanicum* and *Curcuma longa*	Nanoemulsification	Bacterial growth inhibition, methaemoglobin formation (%), lipid oxidation (mgMDAeq/kg), pH levels, colour parameters	CZEO significantly inhibited bacterial growth, reduced metmyoglobin formation, and limited lipid oxidation in minced meat, thereby improving its preservation quality.	• Advantages: Enhanced antimicrobial activity, improved stability, reduced toxicity, masked flavour, homogeneous incorporation into food matrices, effective in maintaining meat quality by inhibiting bacterial growth, reducing methaemoglobin formation, and preventing lipid oxidation. • Disadvantages: Complexity and cost associated with the encapsulation process.	[82]
	Beef	*Syzygium aromaticum* and *Cinnamomum zeylanicum*	Dietary	Lipid oxidation, pH, shear force, meat colour, sensory acceptability, visual acceptability	The CZEO concentration influenced pH, shear force, and meat colour, but did not affect sensory or visual acceptability. The dietary addition of CZEO can reduce lipid oxidation without modifying sensory acceptability attributes.	• Advantages: Affects pH, shear force, and meat colour; reduces lipid oxidation without modifying sensory acceptability. • Disadvantages: Changes in pH, shear force, and meat colour may not always be desirable depending on specific meat quality goals.	[62]
PORK	Meat	*Cinnamomum zeylanicum*	Coating (Kappa-carrageenan-based)	Antimicrobial and antioxidant activities	Microbial growth inhibition, lipid oxidation reduction, desirable pH, and colour persistency were observed throughout refrigerated storage.	• Advantages: Excellent antimicrobial and antioxidant activities. Recognised as safe by regulatory authorities. Effective distribution through edible coatings. Reduced impact on organoleptic characteristics. Effective in preserving meat by inhibiting microbial growth and lipid oxidation. • Disadvantages: Hydrophobicity and instability. Intense aroma limits its application in the food industry.	[85]
	Sausages	*Cinnamomum zeylanicum*	Direct application	Physico-chemical, microbiological, and sensory characteristics	The addition of 0.4 µL/g CZEO had the most effective preservative effect, improving physicochemical, microbiological, and sensory characteristics compared to the control and other essential oil treatment groups.	• Advantages Superior preservation qualities including lower levels of TBARS, free fatty acids, pH, water activity, microbial count, and better sensory properties. • Disadvantages: Not mentioned.	[63]
	Ground meat, Italian-style sausage	*Cinnamomum zeylanicum*	Direct application	Lipid oxidation, instrument colour, total viable aerobic counts, Enterobacteriaceae, biogenic amines, and TVB-N	The 0.5% CZEO treatment was the most effective, improving the microbiological and physicochemical properties of the meat products.	• Advantages Reduced lipid oxidation. Lower microbial counts. Lower biogenic amine contents and TVB-N. Improved colour. • Disadvantages Not mentioned.	[65]
	Meat	*Cinnamomum zeylanicum*	Nanoemulsions	The effect of CZEO nanoemulsions, ε-polylysine (ε-PL), and CZEO/ε-PL on the microbial count (total bacteria counts, *Salmonella*, *Photobacterium*, *Pseudomonas*) and quality attributes (freshness, TVB-N, pH, cooking loss, appearance, odour, texture) of pork during refrigerated storage and radio frequency cooking	The main findings were that CZEO nanoemulsions, both alone and in combination with ε-polylysine (ε-PL), improved the microbial quality and freshness of raw pork during refrigerated storage, and also enhanced the reduction in *Salmonella* and total bacteria counts during radio frequency cooking. The combination of CZEO nanoemulsions and ε-PL also improved the textural properties of the cooked pork.	• Advantages Effective microbial inhibition, improved freshness, reduced cooking time, enhanced bacterial inactivation during cooking, improved textural properties. • Disadvantages Slightly affected odour in radio frequency cooking.	[86]
FISH	Asian seabass (*Lates calcarifer*) fillets	*Cinnamomum zeylanicum*	Nanoemulsions	The CZEO nanoemulsion, bulk CZEO, and sodium hypochlorite antimicrobial activity against foodborne pathogens (*Escherichia coli*, *Salmonella typhimurium*, *Staphylococcus aureus*, and *Vibrio parahaemolyticus*)	CZEO nanoemulsion (11,429 mg/L) was more effective than bulk cinnamon oil in decreasing the initial number of bacteria and inhibiting the pathogens’ growth, especially *Vibrio parahaemolyticus*.	• Advantages It was more effective in reducing the initial number of foodborne pathogens in the fish fillets by 0.5–1.5 log CFU/g. It was more effective in inhibiting the growth of the pathogens, especially *Vibrio parahaemolyticus*, during refrigerated storage. • Disadvantages The nanoemulsion required a higher concentration of CZEO (11,429 mg/L) compared to the bulk oil (488 mg/L), which could impact the cost or feasibility of large-scale application.	[44]

## 5. Regulatory Considerations, Challenges, and Future Perspectives

CZEO underwent rigorous scrutiny with regards to its safety and possible benefits for human health. The Food and Drug Administration (FDA) in the United States supervises the regulation of EOs used in food products, with a main focus on ensuring their safety and stability [87]. The FDA categorises CZEO as GRAS (Generally Recognised As Safe) according to the Code of Federal Regulations (CFR), 2022j [88]. The GRAS designation allows its utilisation without the need for premarket approval, although processors are required to comply with particular requirements outlined in the Federal Food, Drug, and Cosmetic Act, which vary based on the application method and intended purpose. On the other hand, at present, there is no governmental entity in the United States that provides certification or approval for the quality and purity of essential oils [89].

In the European Union, EOs are regulated by laws such as the REACH Regulation (Regulation (EC) No. 1907/2006) and the Classification Labelling and Packaging (CLP) Regulation (EC No. 1272/2008), as well as other regulations that control their legal status [88]. Additionally, the European Food Safety Authority (EFSA) has assessed the utilisation of CZEO in animal feed. For animals with a limited lifespan, it is safe to consume CZEO in drinking water, as long as the total daily consumption does not exceed safe limits (3 mg/L). The EFSA has not found any consumer concerns associated with the use of cinnamon leaf oil and bark oil at these levels [90].

In addition to the legal framework, the principles of Good Manufacturing Practices must be implemented irrespective of the designated EO being utilised. The restrictions also differ based on the particular country where the food product is sold. As an illustration, the European Commission has implemented a policy that is comparable to that of the United States, officially recognising some essential oils. In Japan, the regulation of each form of EO varies [88].

Regarding the hurdles faced, assessing the antimicrobial efficacy of an EO is challenging due to its intricate composition. Several factors, such as the harvesting season and the techniques employed for oil extraction, might influence the makeup of CZEO. Hence, comprehensive data on the CZEO spectrum of susceptible organisms, MIC, mechanism of action, and the impact of food matrix components on antimicrobial qualities can aid in identifying the optimal use in meat products [68,89].

Despite the presence of bioactive components, CZEO’s efficacy is limited due to many factors. These factors include the degradation of compounds in water-based solutions, unpleasant sensory characteristics, interaction with the food matrix (the hydrophobic CZEO compounds are negatively affected by the interactions with fat, starch, and proteins), tendency to evaporate, and susceptibility to oxygen and light [89]. Moreover, its application as a food preservative can be limited due to the requirement of large concentrations to obtain the most effective antimicrobial effect. In order to tackle these challenges, one potential approach is to harness the synergistic effects of more EOs, but there is a lack of research on the interactions that result in synergistic, antagonistic, or additive effects. Conversely, through technological advancements the disadvantages associated with the dose, aroma, and volatility of CZEO could be reduced. In this direction, one approach is to incorporate the EO into active packaging systems. Even so, the modern packaging options still have some drawbacks. For example, there is a proven interplay between certain macromolecules employed as emulsifiers and EO. This interaction might somewhat diminish the antibacterial efficacy of EO. Hence, the identification of an appropriate coating materials represents a crucial aspect to be taken into account [89,91].

Furthermore, given its controlled CZEO release properties, a combination between active packaging methods and conventional ones can be of great interest for the meat industry.

Another CZEO delivery method that can address the aforementioned challenges is encapsulation. Despite its inherent benefits, recent studies suggest that the nanoparticles can penetrate the biological membranes and have cumulative effects in different organs, including liver and brain [92]. Moreover, the use of synthetic nano and microparticle converted wall materials is subjected to intricacies. Higher synthetic wall material concentrations appear to have an impact on the homeostasis balance [93]. In this direction, digestible carriers of natural origin (such as proteins and polysaccharides) can be used to mitigate the concerns [89].

A further major hurdle is that heat processing can lead to a significant reduction in polyphenols, which in turn limits the usage of CZEO in functional foods. In this regard, cinnamaldehyde, a compound with high volatility, is particularly susceptible to degradation when exposed to high temperatures. This degradation leads to the production of undesired by-products such benzene and causes a loss of the original flavour [89,94]. Concerning this aspect, further studies are needed in order to limit this problem.

In addition, the inhibitory action of cinnamaldehyde on yeasts might have a detrimental influence in products that involve fermentation. The food processors must carefully consider the type of product in CZEO functional food formulations [89].

Last but not least, it is crucial to address the costs associated with the addition of CZEO to meat products. The extraction of CZEO in this context necessitates the utilisation of specialised equipment and techniques. Furthermore, the accessibility of the raw material is another related issue that might result in supplementary expenses. The volatility of CZEO can be correlated with specific packaging and storage requirements, which might contribute to the total cost. Nevertheless, scalability poses a further hurdle. Expanding the use of CZEO for mass production could present logistical and economical challenges, such as ensuring uniform quality across large batches. In this direction, further research should comprise cost–benefit analyses to determine the feasibility of large-scale CZEO application.

## 6. Conclusions

Given their composition, meat and meat products can promote the growth of pathogenic and spoilage microorganisms and oxidative reactions. In this direction, cumulative efforts were made to mitigate the associated health hazards and economic downturns. Still, the modern consumer approach tends to revolve around more natural alternatives to synthetic additives. Towards this end, essential oils, serving as clean-label substitutes, can help in carcinogenic prevention and toxic issues associated with synthetic food additives.

The biological activity of CZEO is closely linked to its bioactive compounds, particularly phenolic compounds. These constituents determine the EO’s antimicrobial, antioxidant and health-related benefits. Nevertheless, our superficial understanding regarding the mechanisms that regulate synergy and antagonism is also rooted in the individual essential oil constituents’ mode of action. Numerous investigations have examined the site of action; however, only a handful have proceeded to disclose the mode of action. In this direction, future research should focus more in-depth on their mechanism of action, standardising CZEO formulations, and exploring synergistic effects with other natural preservatives. Moreover, the implementation in the food industry should encompass studies to optimise its use and ensure regulatory compliance and safety.

Furthermore, CZEO can impact the sensory characteristics of meat products. However, studies have shown that such detrimental effects can be alleviated. One example in this direction is the use of edible coatings with encapsulated CZEO to enable its controlled release. Moreover, the use of active packaging materials can reduce the CZEO volatility rate. CZEO can also be used in the vapour phase for indirect contact. Not to be overlooked, the use of nanoemulsion, coating, and film wrapping for controlled release of EOs can pose as promising strategies. Even so, it is worth mentioning that all the application methods have their benefits and drawbacks. The meat industry operators should consider the potential associated challenges before taking into consideration one application method.

In conclusion, CZEO has the potential to contribute to the development of healthier, more sustainable food products. Future research and innovation in this area will pave the way for broader application and greater acceptance.

## Figures and Tables

**Table 2:** Summary of antimicrobial studies on CZEO.

EO	Methodology	CZEO Concentrations	Tested Pathogens	Main Findings	Ref.
*Cinnamomum zeylanicum* bark	Disc diffusion agar, well diffusion agar, cell viability assay, MIC, and MBC methods.	1 to 200 mg/mL	*Escherichia coli*, *Pseudomonas aeruginosa*, *Salmonella typhi*, *Listeria innocua*, *Staphylococcus aureus*, *Bacillus cereus*	CZEO had a strong antibacterial effect on both Gram-positive and Gram-negative bacteria.	[11]
*Cinnamomum zeylanicum* and *Syzygium aromaticum*	Agar disk diffusion assay, MIC, checkerboard method for synergistic activity, growth kinetics studies in buffer suspension and on food, and antibacterial activity assessment on fresh-cut fruits.	1 to 8 µg/mL	*Yersinia enterocolitica*, *Escherichia coli*, *Salmonella Typhimurium*, *Listeria monocytogenes*, *Staphylococcus aureus*	The cinnamon and clove EO demonstrated significant antimicrobial effects against food-borne pathogens, exhibiting a synergistic effect.	[39]
*Cinnamomum zeylanicum* bark	Agar disc diffusion and microdilution broth methods.	0.15–10 μL/mL	Methicillin-resistant *Staphylococcus aureus* (MRSA), vancomycin-resistant *Enterococcus faecium* (VR E. faecium), *Acinetobacter baumannii*, *Pseudomonas aeruginosa*, *Escherichia coli*	All studied extensively drug-resistant isolates were sensitive to CZEO, with MRSA being the most sensitive and *A. baumannii* the least sensitive. The MIC values varied depending on the isolate.	[40]
*Cinnamomum zeylanicum*	MIC, cell viability.	3.3 μL/mL for Gram-positive bacteria and fungi, 10 μL/mL for Gram-negative bacteria	Gram-positive—*Bacillus subtilis* ATCC6633, *Bacillus cereus* ATCC6629 and *Staphylococcus aureus* ATCC29213, Gram-negative—*Escherichia coli* ATCC25922, *Salmonella typhimurium* ATCC14028, *Klebsiella pneumonia* ATCC13883, and *Proteus vulgaris* (isolate) as well as the fungi *Candida albicans* ATCC10231 and *Aspergillus niger*	CZEO showed highly significant antimicrobial activity against all tested pathogens.	[31]
*Cinnamomum zeylanicum*	In vitro release analysis and comparative antibacterial activity tests.	Nanoparticles yield approx. 55–72% *w*/*w*	*Escherichia coli*, *Erwinia carotovora*, *Pseudomonas fluorescens*	The study found that encapsulating CZEO in chitosan nanoparticles significantly enhanced its antibacterial activity, with *Pseudomonas fluorescens* being more sensitive to the encapsulated EO. The highest antibacterial activity was observed against *E. coli*.	[41]
*Cinnamomum zeylanicum*	The determination of the viable cells and bacterial biomass quantification.	0.0%, 0.12%, 0.48%, 0.96%, 1.92%	*Escherichia coli*, *Staphylococcus aureus*	The 1.92% CZEO concentration was effective in reducing *Escherichia coli* by 5.91 log CFUcm^−2^ and *Staphylococcus aureus* by 5.17 log CFUcm^−2^, indicating its potential as an antibiofilm agent.	[42]
*Cinnamomum zeylanicum*	In vivo methods (e.g., supplementation in broiler diets to observe changes in caecal microbiota) and in vitro methods (e.g., MIC and MBC values). Specific tests included measuring the inhibitory effect on bacterial growth and the reduction in bacterial counts.	200 mg/kg and 500 mg/kg	*Parahemolyticus*, *Staphylococcus epidermis*, *Enterococus faecalis*, *Pseudomonas aeruginosa*, *Salmonella* sp., *Staphylococcus aureus*, *Escherichia coli*, *Campylobacter jejuni*	The EO had antimicrobial effects on the tested pathogens.	[37]
*Cinnamomum zeylanicum* (bark and leaf)	Disc diffusion and minimum inhibitory concentration assay.	MIC = 5 μL/mL (bark, *Staphylococcus aureus*), MIC = 2.5 μL/mL (bark, *Aspergillus niger*), MIC = 5 μL/mL (leaf, *Bacillus cereus*), MIC = 2.5 μL/mL (leaf, *Aspergillus niger*)	*Staphylococcus aureus*, *Aspergillus niger*, *Bacillus cereus*	Both the bark and leaf CZEO of Blume exhibited good antimicrobial properties.	[43]
*Cinnamomum zeylanicum*	Compared reduction in bacterial count (log CFU/g) and inhibition of bacterial growth in artificially contaminated refrigerated Asian seabass fillets treated with different concentrations of CZEO nanoemulsion, bulk cinnamon oil, and sodium hypochlorite.	1429 mg/L and 11,429 mg/L	*Escherichia coli*, *Salmonella Typhimurium*, *Staphylococcus aureus*, *Vibrio parahaemolyticus*	The nanoemulsion formulation of CZEO significantly enhanced its antimicrobial activity against foodborne pathogens in refrigerated Asian seabass fillets, particularly reducing bacterial counts by approximately 0.5–1.5 log CFU/g. CZEO was more effective than bulk cinnamon oil and sodium hypochlorite, especially against *Vibrio parahaemolyticus*.	[44]
*Cinnamomum zeylanicum*	In vitro evaluation of antimicrobial activity and MIC values.	6.25%, 3.12%, and 3.12% (*v*/*v*)	*Staphylococcus aureus*, *Escherichia coli*, *Salmonella enterica*	CZEO showed the lowest MIC values for the tested pathogens. A subinhibitory concentration was able to inhibit the adhesion of these pathogens to polystyrene surfaces.	[45]

**Table 3:** Summary of antioxidant studies on CZEO.

EO	Methodology and Results	Conclusion	Ref.
*Cinnamomum zeylanicum* bark	DPPH radical scavenging, which showed 71.12 ± 0.77% activity β-carotene bleaching assay, which showed 63.08 ± 0.81% inhibition	CZEO has strong antioxidant properties demonstrated by its ability to scavenge free radicals and inhibit lipid oxidation.	[11]
*Cinnamomum zeylanicum*	Hydrogen peroxide scavenging assay—30.73% activity Nitric oxide scavenging assay—15.23% activity	The CZEO extracted from the bark has antioxidant properties.	[27]
*Cinnamomum zeylanicum Blume bark and leaf essential oils*	DPPH free radical scavenging assay (half-maximal inhibitory concentration—IC_50_ = 103.2 μg/mL for the bark essential oil and 234.7 μg/mL for the leaf essential oil) and reducing power assay (absorbance of 1.802 nm and 0.907 nm in 48 μg/mL for bark and leaf CZEO)	The bark and leaf CZEO of Blume have antioxidant properties, with the bark essential oil showing a stronger antioxidant activity than the leaf essential oil.	[43]
*Cinnamomum zeylanicum Blume essential oil*	Phosphomolybdenum assay (108.75 ± 32.63 mg of essential oil/equivalent to 1 mg of vitamin C) DPPH radical scavenging assay (21.3%) Hydrogen peroxide scavenging assay (55.2%)	CZEO of Blume has significant antioxidant properties demonstrated by its ability to scavenge DPPH and hydrogen peroxide radicals, as well as its high antioxidant capacity equivalent to vitamin C.	[12]

## Data Availability

The original contributions presented in the study are included in the article, further inquiries can be directed to the corresponding author.
